# Endoscopic Advancements in Pediatric Pancreatitis

**DOI:** 10.3389/fped.2022.937136

**Published:** 2022-06-15

**Authors:** Michelle Saad, David S. Vitale

**Affiliations:** ^1^Division of Gastroenterology, Hepatology and Nutrition, Cincinnati Children's Hospital Medical Center, Cincinnati, OH, United States; ^2^Department of Pediatrics, University of Cincinnati College of Medicine, Cincinnati, OH, United States

**Keywords:** endoscopy, interventional endoscopy, EUS, ERCP, pancreatitis, pediatric

## Introduction

Acute pancreatitis (AP) in children occurs with an estimated annual incidence of 3–13/100,000 (1, 2). While some children may have a single episode, others may develop acute recurrent pancreatitis (ARP) or chronic pancreatitis (CP). Children with pancreatic disease can have impacted quality of life due to chronic pain, frequent hospitalizations, and/or nutritional deficiencies. Supportive management is typically indicated for patients with acute pancreatitis, while in some circumstances endoscopic diagnostic evaluation and/or therapy are required. Endoscopy can also be beneficial in patients with ARP or CP (3).

Historically, interventional endoscopy procedures which can benefit patients with pancreatitis, such as endoscopic retrograde cholangiopancreatography (ERCP) and endoscopic ultrasonography (EUS), have been performed by adult gastroenterologists with advanced endoscopic training. Adult physicians certainly have adequate training to perform the procedures, but they often lack formal training in caring for pediatric patients or pediatric pancreatitis. Many are employed in facilities that care exclusively for adult patients and either travel to pediatric institutions to perform procedures in an unfamiliar setting or perform procedures for pediatric patients in adult facilities. This approach, when executed well, can be very successful and provide excellent patient care. However, issues can arise utilizing this model due to limited physician availability, timeliness of care and procedures, and challenges in communication amongst pediatric providers, adult proceduralists and families. In an ideal setting, children should be treated at a center with dedicated pediatric nursing staff, anesthesia, behavioral and child life specialists, and pediatric surgical and intensive care expertise if needed (4).

Over the last 10–15 years, pediatric gastroenterologists have increasingly pursued training in interventional endoscopy. Pediatric ERCP, EUS, and other advanced endoscopic procedures are now performed safely and effectively in specialized centers by pediatric providers worldwide (5–7). New training opportunities for pediatric gastroenterologists in interventional endoscopy continue to arise (8). In conjunction with advancements in interventional endoscopy, the field of pediatric pancreatology continues to evolve through increased recognition of AP and CP and collaborative approaches to research (9–11). It is increasingly important that providers managing children with pancreatitis are aware of the indications for endoscopic evaluations and interventions for pancreatitis, the benefits and risks involved, and when endoscopic therapy is no longer warranted (3).

## Endoscopic Ultrasound

Endoscopic ultrasonography allows for highly detailed transgastric and transduodenal sonographic images of the pancreas and peripancreatic anatomy through an echoendoscope and can be safely and effectively performed in children (3, 6, 12, 13). EUS has been established in adults since the 1980s and is sensitive for evaluating changes in the pancreas that reflect CP, including specific parenchymal and ductal changes. While traditional cross-sectional imaging often reflects late irreversible changes to the pancreas, EUS can allow the detection of early changes or minimal change CP before irreversible changes occur (14). EUS has been shown to outperform cross-sectional imaging [magnetic resonance imaging (MRI) and computed tomography (CT)] for the detection of CP with a sensitivity of 81% and specificity of 90% (15). To maximize the success of interventions aimed at slowing or stopping disease progression and ameliorating the deleterious downstream effects of pancreatic insufficiency, techniques to identify the early stages of chronic pancreatic disease are imperative.

In adult patients, Rosemont or conventional criteria are used to assess CP parenchymal or ductal changes (14, 16–18). These guidelines are utilized but not accepted universally in adults, with poor reliability amongst different observers (17). These established criteria can be utilized as a guide in assessing the pediatric pancreas *via* EUS, however results in pediatric patients must be interpreted with caution as there is no data to validate its use in children (14, 16, 19).

Endoscopic ultrasonography can also be used in patients with idiopathic ARP or CP, investigating for ductal or anatomic abnormalities not fully delineated by magnetic resonance cholangiopancreatography (MRCP), or even biliary microlithiasis. EUS identifies an etiology in up to 75% of patients with idiopathic ARP (20). More recent innovations in EUS diagnostic imaging include the use of real-time shear wave elastography. EUS elastography allows an indirect assessment of tissue rigidity and may predict pancreatic fibrosis or pancreatic exocrine insufficiency (21, 22). Contrast-enhanced EUS consists of EUS imaging while gas-filled microbubbles are injected into peripheral veins, highlighting vascular lesions within the pancreas and helping distinguish various pancreatic lesions. In benign pancreatitis, contrast-enhanced EUS can identify necrotizing foci of AP at an early stage and may be useful in differentiating focal autoimmune pancreatitis from pancreatic cancer (23, 24).

Autoimmune pancreatitis (AIP) presents a challenging diagnostic dilemma, with convoluted adult diagnostic criteria which include response to therapy (25). Pediatric guidelines from the International Study Group of Pediatric Pancreatitis: In Search for a Cure (INSPPIRE) suggest “tissue diagnosis should ideally be obtained prior to initiating therapy,” with EUS guided biopsies favored when available (26). Diagnostic sensitivity for EUS guided fine needle biopsy (FNB) is quite low in adults (27). Pediatric patients typically present with type 2, rather than type 1 AIP and there is a paucity of data in children (26, 28). A recent meta-analysis showed the pooled diagnostic yield for histology criteria in AIP to be 55.8% for FNA and 87.2% for FNB despite similar rates of histologic tissue procurement (29). In our practice, cases of suspected autoimmune pancreatitis with classic, diffuse imaging findings are often treated empirically after discussing risks/benefits with the family. Cases that are less clear benefit from extensive discussions with families regarding the typical modest yield of EUS-FNB, along with the risks and benefits of empiric therapy vs. biopsy.

Interventional EUS is a rapidly progressing field utilizing echo endoscopes to perform therapeutic interventions and should be performed at high volume, experienced centers. The predominant use of interventional EUS in pediatric patients is in symptomatic pancreatic walled off necrosis and pseudocysts. EUS-guided transgastric or transduodenal cyst-enterostomy drainage procedures are preferred approaches with superior success rates and risk profiles, decreased length of stay and lower treatment cost as compared to surgical approach (3, 30, 31). Metal and plastic stents have been used in children successfully, along with lumen apposing metal stents (13, 32–34). EUS guided celiac plexus blockade in patients with CP and debilitating pain has been used successfully in adult and pediatric patients (6, 35–37). Pediatric patients with conventional or post-surgical anatomy with dilation of the pancreatic duct but the inability to cannulate the duct *via* ERCP are candidates for pancreatic duct rendezvous *via* EUS (38).

The risk of adverse events in EUS includes bleeding, bacteremia and perforation. FNA or FNB of the pancreatic parenchyma can cause pancreatitis. Diagnostic EUS presents risk rates similar to upper endoscopy, while interventional EUS presents higher risks of infection, bleeding and perforation. The failure rate for complex procedures, such as pancreatic rendezvous, can approach 30% (6, 39, 40).

## Endoscopic Pancreatic Function Testing

Exocrine pancreatic insufficiency (EPI) can occur in patients with cystic fibrosis and other congenital diseases, and CP. Indirect EPI testing is available, but only detects severe EPI. Direct pancreatic function testing with endoscopic pancreatic function testing (ePFT) during conventional esophagogastroduodenoscopy has emerged as a viable test in children. The North American Society of Pediatric Gastroenterology, Hepatology and Nutrition (NASPGHAN) recently published a position paper outlining a proposed standardized ePFT protocol in children. After aspiration of gastric and duodenal contents, cholecystokinin or secretin are administered and 3 duodenal aspirates are collected at 5-min intervals and sent for laboratory analysis of pancreas enzyme activity. EUS with secretin stimulation (sEUS) has been utilized to assess for structural changes reflective of minimal change chronic pancreatitis (MCCP), and can impact the likelihood of CP following, and predict progression of MCCP to overt CP in patients with abdominal pain thought to be pancreatic in origin with non-diagnostic cross-sectional imaging (41, 42). A multicenter research collaboration is needed to further refine and validate the proposed methods (43).

## Endoscopic Retrograde Cholangiopancreatography

Acute recurrent pancreatitis and CP are some of the most frequent indications for pediatric ERCP, which is feasible in children of all ages and sizes (3, 7, 44, 45). Historically diagnostic ERCP had been utilized, however, with the advent of high quality MRCP and EUS, the vast majority of pediatric patients undergo ERCP for therapeutic indications (7). ERCP is used sparingly for diagnostic purposes, however in cases of suspected pancreatic ductal anatomic abnormalities, such as pancreas divisum, anomalous pancreaticobiliary junction or choledochocele it remains the gold standard (3, 46, 47). Diagnoses of abnormal pancreaticobiliary ductal anatomy can be confirmed and sometimes treated within the same procedure.

Endoscopic retrograde cholangiopancreatography allows numerous therapeutic modalities for ARP and CP in symptomatic patients. Pancreatic duct strictures are managed with catheter or balloon dilation, followed by serial pancreatic duct (PD) stent placements for up to 12 months with close observation (48). Calculi in the PD can also be removed using an extraction balloon during ERCP or for larger refractory stones through extracorporeal shockwave lithotripsy or pancreatoscopy (49). Other anatomical abnormalities such as pancreas divisum associated with ARP or CP can be addressed through a minor papillotomy and/or dorsal pancreatic duct stent placement (50–52).

Pediatric patients with AP may benefit from ERCP as well. ERCP with stent placement may be necessary for AIP with associated pancreatic or bile duct strictures/obstruction. Therapeutic intervention through biliary sphincterotomy and stone extraction is performed if AP is due to biliary etiology, specifically gallstone pancreatitis with suspected persistent bile duct obstruction (53, 54).

Technically successful ERCP may not always alleviate patients' symptoms in the setting of pancreatic disease. Moreover, ERCP is associated with certain risks, including post ERCP pancreatitis (PEP), bleeding, infection, and perforation (5). PEP occurs in up to 12% of children undergoing ERCP and is more likely to occur in patients needing pancreatic sphincterotomy, PD cannulation, PD injection, or prophylactic stent placement (55, 56). Pre-emptive use of IV ketorolac or ibuprofen in children has been performed at the time of ERCP to decrease the rates of PEP, albeit published results have not reached statistical significance (55, 57).

## Surgical Management

Patients who do not respond to endoscopic therapy and have progressive, debilitating pancreatitis, may require surgical intervention. Traditional surgical drainage procedures such as lateral pancreaticojejunostomy (Puestow), or other surgical drainage variants (Frey or Beger), pancreatic tail resection, or pancreaticoduodenectomy (Whipple) have fallen out of favor in children with ARP and CP, especially in those with genetic risk factors. Endoscopic procedures can improve drainage in cases of pancreatic duct strictures amenable to stenting, usually attempted for up to a year before surgical intervention (48).

Children debilitated by their disease, with chronic pain, frequent hospitalizations, and failure of maximized medical and endoscopic therapy can be considered for total pancreatectomy with islet auto-transplantation (TPIAT) after an extensive evaluation. Total pancreatectomy offers the advantage of pain relief but leads to diabetes. The risk of diabetes is offset by the islet auto-transplantation intraoperatively, with childrens' glycemic outcomes ranging from insulin-independent to diabetic, with contributing factors such as the timing of surgery from the onset of symptoms, body mass index, pancreas mass, and fibrosis (58). Pancreatic enzyme replacement therapy is also needed post-operatively as patients have acquired exocrine pancreatic insufficiency.

## Pediatric Interventional Endoscopy: The Future

While the field of pediatric interventional endoscopy has evolved, many care gaps exist and there are tremendous opportunities for growth and research (3, 45). Awareness of available pediatric diagnostic and interventional procedures will need to continue to increase. Unique research opportunities arise in pediatric pancreatitis, with the ability to study the progression and entire spectrum of disease by following patients from a first episode of AP to CP. EUS findings along the spectrum of disease, including objective findings of shear wave elastography, could lead to the development of a pediatric EUS CP criteria (59).

Endoscopic retrograde cholangiopancreatography and EUS are frequently used to diagnose and treat autoimmune pancreatitis and pancreas divisum, but supporting literature is sparse. Individualized decisions regarding endotherapy related to specific genetic mutations needs further research and randomized studies are needed to further assess PEP prophylaxis.

The volume of interventional endoscopic procedures in children is increasing, however it still does not approach adult volume. Strong collaboration amongst institutions and individual endoscopists remains vital to the advancement of the field. As the field evolves, questions remain regarding appropriate training avenues and the appropriate location and number of centers offering pediatric interventional endoscopic expertise.

## Conclusion

Increased awareness of pancreatic disease in children has led to improved detection of AP, ARP, and CP in this population. Diagnostic and therapeutic options with EUS and ERCP are available and ideally should be performed at large tertiary centers with high patient volumes. The approach to each patient is individualized, and some may not need endoscopic diagnostic or therapeutic intervention. Surgical options such as TPIAT should be considered in patients with debilitating pain and affected quality of life who also need serial endoscopic therapy with no notable improvement in symptoms or frequency of inflammatory attacks. There are ample research opportunities to advance the fields of pediatric interventional endoscopy and pancreatology as they continue to evolve.

## Author Contributions

MS and DV contributed to the conceptualization, drafting of the manuscript, and editing of the final manuscript. Both authors contributed to the article and approved the submitted version.

## Conflict of Interest

The authors declare that the research was conducted in the absence of any commercial or financial relationships that could be construed as a potential conflict of interest.

## Publisher's Note

All claims expressed in this article are solely those of the authors and do not necessarily represent those of their affiliated organizations, or those of the publisher, the editors and the reviewers. Any product that may be evaluated in this article, or claim that may be made by its manufacturer, is not guaranteed or endorsed by the publisher.
